# Single Molecule Studies of the Diffusion of Band 3 in Sickle Cell Erythrocytes

**DOI:** 10.1371/journal.pone.0162514

**Published:** 2016-09-06

**Authors:** Jeff Spector, Gayani C. Kodippili, Ken Ritchie, Philip S. Low

**Affiliations:** 1 Department of Physics, Purdue University, West Lafayette, IN, 47907, United States of America; 2 Department of Chemistry, Purdue University, West Lafayette, IN, 47907, United States of America; Institut national de la santé et de la recherche médicale - Institut Cochin, FRANCE

## Abstract

Sickle cell disease (SCD) is caused by an inherited mutation in hemoglobin that leads to sickle hemoglobin (HbS) polymerization and premature HbS denaturation. Previous publications have shown that HbS denaturation is followed by binding of denatured HbS (a.k.a. hemichromes) to band 3, the consequent clustering of band 3 in the plane of the erythrocyte membrane that in turn promotes binding of autologous antibodies to the clustered band 3, and removal of the antibody-coated erythrocytes from circulation. Although each step of the above process has been individually demonstrated, the fraction of band 3 that is altered by association with denatured HbS has never been determined. For this purpose, we evaluated the lateral diffusion of band 3 in normal cells, reversibly sickled cells (RSC), irreversibly sickled cells (ISC), and hemoglobin SC erythrocytes (HbSC) in order to estimate the fraction of band 3 that was diffusing more slowly due to hemichrome-induced clustering. We labeled fewer than ten band 3 molecules per intact erythrocyte with a quantum dot to avoid perturbing membrane structure and we then monitored band 3 lateral diffusion by single particle tracking. We report here that the size of the slowly diffusing population of band 3 increases in the sequence: normal cells<HbSC<RSC<ISC. We also demonstrate that the size of the compartment in which band 3 is free to diffuse decreases roughly in the same order, with band 3 diffusing in two compartments of sizes 35 and 71 nm in normal cells, but only a single compartment in HbSC cells (58 nm), RSC (45 nm) and ISC (36 nm). These data suggest that the mobility of band 3 is increasingly constrained during SCD progression, suggesting a global impact of the mutated hemoglobin on erythrocyte membrane properties.

## Introduction

Sickle cell disease is an inherited red blood cell (RBC) disorder that arises from the mutation of the 6^th^ amino acid in the beta chain of hemoglobin (Hb) from glutamic acid to a valine [1], promoting polymerization of the mutated Hb under hypoxic conditions and premature denaturation of the protein during circulation. This accelerated denaturation of sickle hemoglobin (HbS) leads to formation of hemichromes in which the protein still binds iron, albeit in its oxidized state, but can no longer bind oxygen. Importantly, hemichromes exhibit an increased affinity for the NH_2_-terminus of band 3, inducing clustering of the anion transport protein in the plane of the erythrocyte membrane [2, 3], which upon further propagation causes collection of the hemichromes-band 3 clusters into macroscopic aggregates termed Heinz bodies [4, 5]. These microscopic and macroscopic aggregates of band 3 trigger the binding of an autologous anti-band 3 antibody that can either promote premature removal of the affected RBC or cause the “pitting” of the aggregate from the red cell surface in the spleen, releasing an RBC with reduced membrane surface area back into circulation [6]. In addition to causing the premature removal and decrease in surface to volume ratio of the sickle cell, the HbS mutation leads via unknown mechanisms to abnormal cation homeostasis, lipid bilayer dysfunction, intravascular hemolysis, and unwanted adhesion of the aberrant RBC to the vascular endothelium [7]. These changes in erythrocyte properties can result in vaso-occlusion and intravascular thrombosis, leading to the painful crises and organ failure characteristic of the disease [8].

While many aspects of the above chronology of events have been well documented, little information is available on the fraction of band 3 molecules that are impacted by the binding of denatured HbS to the membrane. An excellent study comparing the diffusion of band 3 in sickle and normal RBCs of different densities has revealed that the rotational and lateral diffusion of band 3 becomes increasingly restricted as the density of the sickle and normal cell populations increase [9]. The authors also demonstrate that the diffusion of glycophorin A is similarly constrained in cells of increasing density [9]. However, because these studies employed methods that measure the average diffusion of the entire population of band 3 molecules, it was difficult to determine what fraction of band 3 were affected by binding of denatured HbS to the membrane.

In an effort to quantify the diffusion of individual band 3 molecules, we have developed a DIDS-biotin conjugate that binds very specifically to band 3 and allows for tracking of single band 3 molecules when used in conjunction with a streptavidin-linked quantum dot [10]. We have previously reported that ~¼ of the band 3 molecules in normal RBCs are free to diffuse in an unconstrained manner, with the remainder exhibiting more restricted diffusion characterized by microscopic diffusion coefficients (D_μ_) ranging from 10^−9^ to 10^−13^ cm^2^/s (median D_μ_ = 1.4x10^-11^) [10]. The latter more constrained population has been further shown to be comprised of two overlapping subpopulations, namely, a very slowly diffusing subset of band 3 molecules bound to adducin at the junctional complex and a more rapidly (but still moderately immobilized) subset bound to ankyrin at the ankyrin complex [11]. In the following study, we have utilized the same single molecule tracking techniques to monitor the diffusion of individual band 3 molecules in otherwise unlabeled intact normal and sickle erythrocytes (i.e. reversibly sickled cells, irreversibly sickled cells and HbSC cells). We report here that all populations of band 3 diffuse more slowly in cells expressing HbS and that this diffusion decreases according to the sequence: normal RBCs>HbSC>RSC>ISC.

## Methods

### Labeling red blood cells

After receipt of written informed consent from healthy volunteers or patients with sickle cell disease, blood was voluntarily donated for the purpose of this study in accordance with institutional review board approval from the cooperating medical centers (University of Illinois at Chicago and Children’s Hospital Oakland) and Purdue University. Normal RBCs were defined as those from healthy volunteers which exhibited no obvious clinical symptoms or abnormalities in erythrocyte morphology or stability. All patients expressing sickle hemoglobin (HbSS and HbSC) exhibited sickle cell disease symptoms at the time of blood collection. The methodology for processing samples from both healthy and sickle cell donors followed the same previously published method [10]. In brief, blood was collected into anticoagulant tubes (BD Vacutainer® ACD /Solution B) and shipped overnight to Purdue University for analysis. Blood was centrifuged the next morning at 1000 x g to pellet erythrocytes, after which the red cells were removed and washed 3x in PBS containing 5 mM glucose. RBCs were diluted to 5% hematocrit in PBS/glucose buffer incubated with 10^−11^ M DIDS-biotin at 37°C for 1.5h in order to derivatize fewer than 10 band 3 molecules per RBC. DIDS-biotin labeled cells were then incubated at room temperature with streptavidin Qdot 525. Unbound streptavidin conjugates were removed by washing twice with 0.1% BSA in PBS. Labeled cells were allowed to settle onto a pre-cleaned, poly-lysine-coated cover slip. Finally, 500 μL of PBS were added to the chamber and the cells were imaged as described previously [10].

### Single quantum dot fluorescence video microscopy

**Fig 1:**
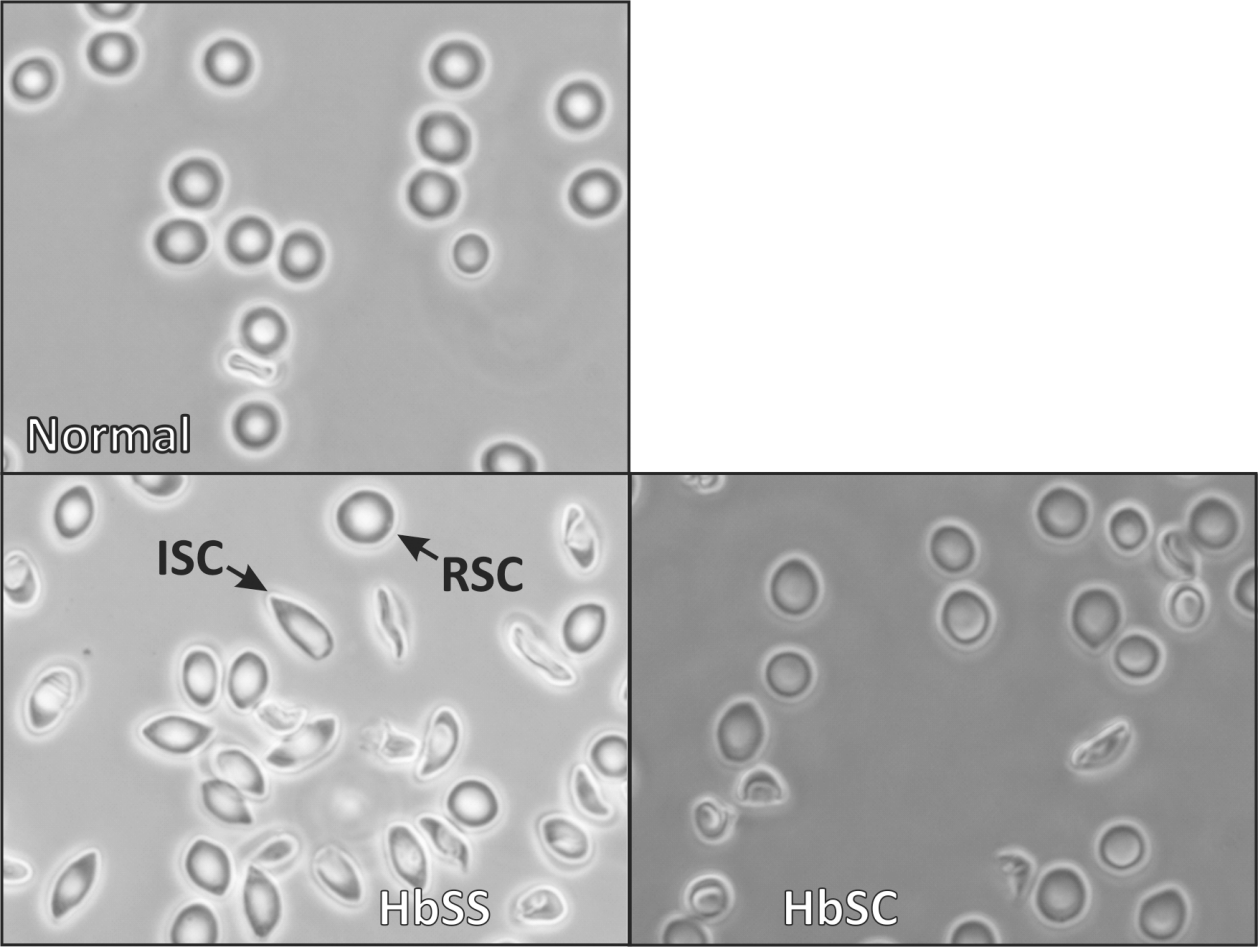
Representative differential interference contrast microscope images of erythrocytes examined in this study. Normal erythrocytes, erythrocytes from an HbSS patient with both reversibly sickled cells (RSC) and irreversibly sickled cells (ISC), and HbSC erythrocytes are shown in separate panels.

The protocol for the imaging and video microscopy follows the previously published and detailed method described by Kodippili at al. [10]. Briefly, oblique illumination by a 488 nm Argon ion laser was used to excite the quantum dots. The quantum dot (qdot) emission was filtered through a 525/50 band pass filter and incident upon a dual MCP intensified cooled CCD camera (XR/Turbo-120z, Stanford Photonics, Inc., Palo Alto, CA). Only data from quantum dots on the upper surface of the cells was recorded to remove any artifacts created by the poly-lysine layer. Individual qdots were recorded for 1000 frames at 120 frames/second (8.333 ms/frame). Due to the stochastic nature of quantum dot blinking only quantum dots that were observed for at least 40 consecutive frames were considered for further analysis. The qdot trajectories were collected from a random selection of RBCs in each sample. After each recording a bright field image was acquired to allow us to distinguish between ISC and RSC based on cell morphology (Fig 1). All imaging was performed on a temperature controlled stage that was maintained at 37°C.

### Analysis of band 3 mobility

The position of the quantum dot in the video image was determined using the algorithm described by Gelles et al. [12]. First, the image of the qdot was cross-correlated with a 2-D Gaussian kernel of a defined width and amplitude. Then a threshold was applied to the cross-correlated image and the center-of-mass of the image was found. This was taken to be the position of the qdot. This process was repeated for as long as the qdot was visible, giving a trajectory that represents the position of the qdot as a function of time. A more detailed description of this analytical technique was published previously [10]. The resulting distributions of diffusion coefficients were then plotted on a Log scale and a Gaussian fit was applied to determine the mean value of the distribution. Each distribution was fit with single and bimodal distributions and an F-test at a 95% confidence interval was used to determine the correct number of populations.

## Results

### Band 3 dynamics in reversibly sickled erythrocytes

**Fig 2:**
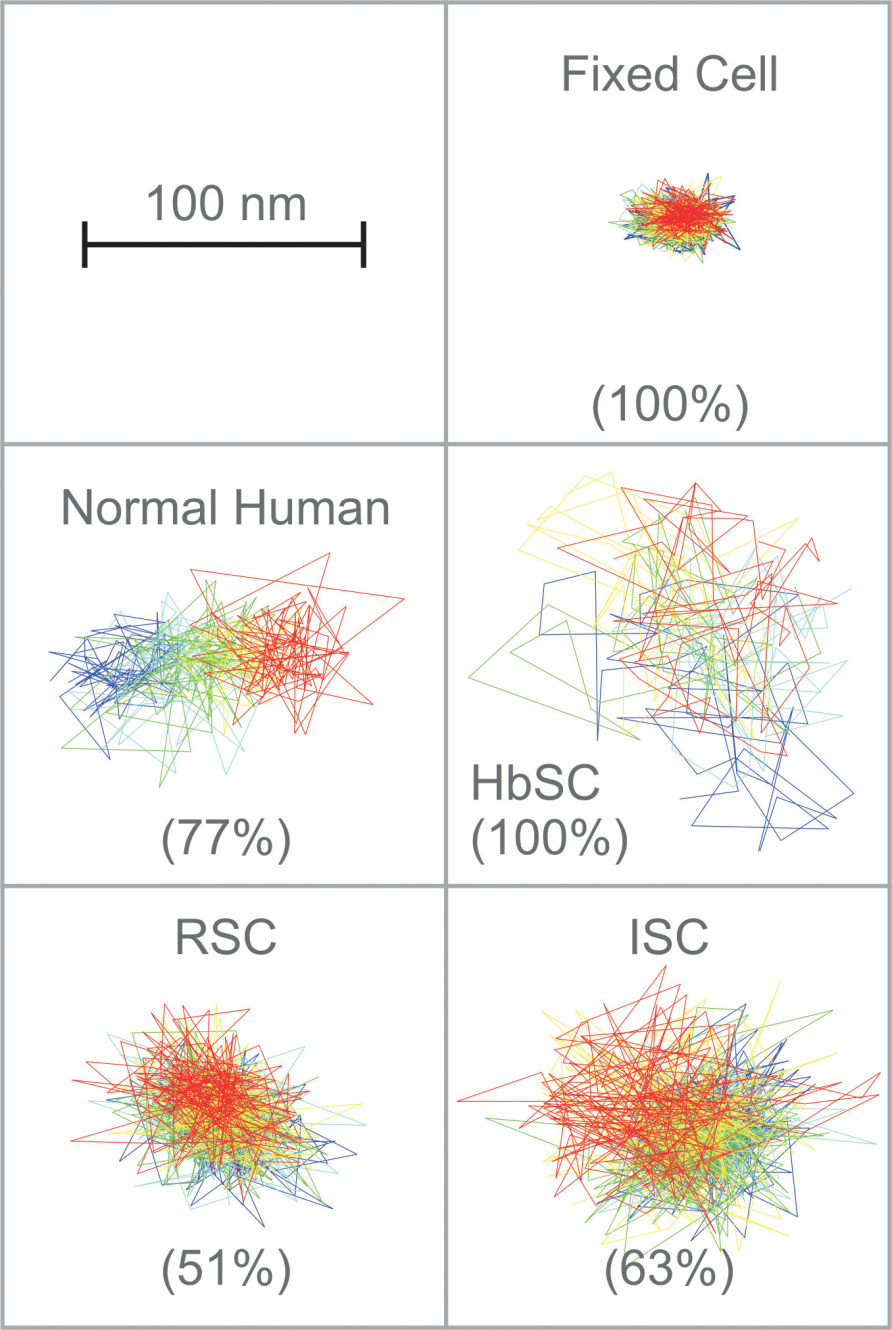
Representative trajectories of DIDS-biotin labeled band 3 on intact normal and sickle human erythrocytes. After labeling with DIDS-biotin conjugate, the diffusion of the labeled band 3 was imaged for 100 consecutive frames at 120 frames/sec on intact fixed normal red blood cells, unfixed normal erythrocytes, HbSC erythrocytes, reversibly sickled cells (RSC) or irreversibly sickled cells (ISC). The different colors track band 3 diffusion as a function of time, beginning with blue and progressing through the colors of the rainbow to red. Because all of these erythrocytes have multiple subpopulations of band 3, the trajectory of only the most abundant population is displayed (% of total band 3 is indicated in parentheses).

**Fig 3:**
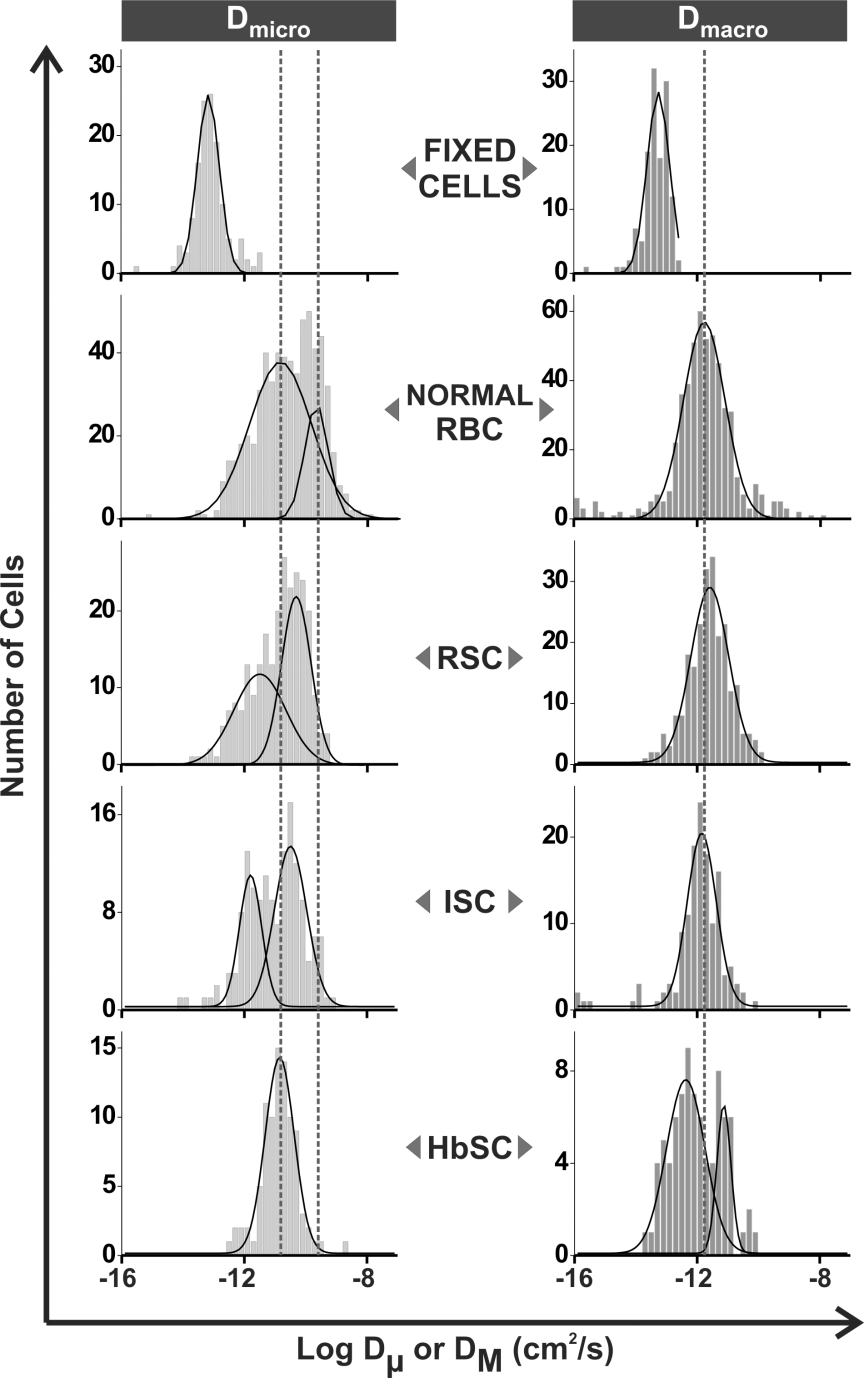
Distributions of the logarithms of the microscopic (D_μ_) and macroscopic (D_M_) diffusion coefficients of band 3 in healthy and sickle erythrocytes. Diffusion coefficients were determined by analysis of individual trajectories of labeled band 3 molecules in intact fixed normal cells, unfixed normal cells, reversibly sickled cells (RSC), irreversibly sickled cells (ISC), and HbSC erythrocytes.

Representative images of normal, HbSS erythrocytes (reversible and irreversible), and HbSC erythrocytes are shown in Fig 1. A typical trajectory of band 3 on intact reversible sickle cells (RSC) is shown in Fig 2. Analysis of the trajectories of band 3 (n = 259 from 7 individual donors) in RSC samples yielded a distribution of microscopic diffusion coefficients (D_μ_) which fits well within two Gaussian curves. Per Fig 3 and Table 1, approximately half of the population centered at 3.2×10^−12^ cm^2^/s (i.e. 5x slower than the faster population in normal RBCs with a mean of 2.2×10^−10^ cm^2^/s [23%]) and the other half of the population centered at 4.9×10^−11^ cm^2^/s (i.e. 4x slower than the slow population in normal RBCs with a mean of 1.4×10^−11^ cm^2^/s [77%]). This microscopic diffusion data suggests that the band 3 diffusion is somehow impeded. In contrast, the macroscopic diffusion coefficient (D_M_) displays a single broad peak with mean value of 2.6×10^−12^ cm^2^/s (Fig 3, Table 1); i.e. similar to normal RBCs with a mean D_M_ of 1.7×10^−12^ cm^2^/s (for individual patient diffusion data see S1 Fig). The D_M_ is generally slower than D_μ_ due to interactions with the cytoskeleton [13, 14], although approximately 10% of the D_M_ data is ≥ the mean of the lower of the two D_μ_. This may be the case for band 3 that is tethered via either ankyrin or adducin to the spectrin-based cytoskeleton.

**Table 1:** Microscopic and macroscopic diffusion coefficient data for various healthy and sickle cell erythrocytes populations.

RBC	D_μ_, (cm^2^/s)	D_M_, (cm^2^/s)
Fixed (n = 134)*	(6.8 ± 0.1) × 10–14	(5.5 ± 0.2) ×10–14
Normal (n = 626)	(1.4 ± 0.4) × 10–11 [77%]**	(1.7 ± 0.1) × 10–12
	(2.2 ± 0.1) × 10–10 [23%]	
RSC (n = 259)	(3.2 ± 2.5) × 10–12 [49%]	(2.6 ± 0.1) × 10–12
	(4.9 ± 0.5) × 10–11 [51%]	
ISC (n = 151)	(1.6 ± 0.2) × 10–12 [37%]	(1.4 ± 0.1) × 10–12
	(3.2 ± 0.3) × 10–11 [63%]	
HbSC (n = 90)	(1.4 ± 0.6) × 10–11	(4.3 ± 0.4) × 10–13 [76%]
		(7.4 ± 0.5) × 10–12 [24%]

Data are means ± standard error of the mean. RBC: red blood cell; RSC: reversibly sickled cells; ISC: irreversibly sickled cells

* Total number of trajectories analyzed.

** Reflects the fraction of total area contained in each peak of the Gaussian fit to the distribution. For those distributions that fit best with a single peak it is understood that 100% of the area is contained within that peak

### Band 3 dynamics in irreversibly sickled erythrocytes

A typical trajectory of band 3 on an intact irreversibly sickled cells (ISC) is shown in Fig 2. Analysis of the trajectories of band 3 (n = 151 from 5 individual donors) in ISC samples at 37°C revealed two Gaussian distributions for D_μ_ centered at 1.6×10^−12^ cm^2^/s (i.e. 9x slower than the slow population in normal RBCs and 2x slower than the slow population in RSC; Fig 3, Table 1) and 3.2×10^−11^ cm^2^/s (i.e. 7x slower than the faster population in normal RBCs). In contrast to normal RBCs, both fast and slow populations of D_μ_ of ISCs are two times slower than D_μ_ of RSC. The macroscopic diffusion of ISC samples (for individual patient diffusion data see S2 Fig) exhibited a single Gaussian peak, similar to normal D_M_, centered at 1.4×10^−12^ cm^2^/s. Thus D_M_ of ISC is two times slower than D_M_ of RSC. In short, ISC microscopic and macroscopic data suggests that band 3 diffusion is impeded by more barriers in ISC than RSC.

### Band 3 dynamics in HbSC erythrocytes

Analysis of the trajectories of band 3 (n = 90 from 5 individual donors) in intact HbSC cells (Fig 2) revealed a microscopic diffusion coefficient characterized by a single Gaussian distribution with a mean D_μ_ value of 1.4x10^-11^ cm^2^/s (Fig 3, Table 1). This diffusion coefficient is similar to the D_μ_ of the slow population in normal RBC. The distribution of macroscopic diffusion coefficients showed two peaks centered at 4.3×10^−13^ (76%) and 7.4×10^−12^ cm^2^/s (24%) (Fig 3, Table 1). This major macroscopic population was moving 3x slower than that found in normal red blood cells, while the remaining 24% was diffusing slightly faster. Interestingly, HbSC cells displayed a bimodal distribution in the long time diffusion coefficient (D_M_), where the faster population appeared to actually be displaying diffusion behavior similar to what is observed on normal cells, while the majority of band 3 (75%) were diffusing approximately 10-fold slower than what was seen in normal, ISC and RSC RBCs. These data would imply that on longer timescales the diffusion of the majority of band 3 is more restricted in HbSC cells than in either of RSC or ISC cells. This could be the case if, for example, a band 3 molecule were confined to a corral where it could exhibit rapid short time diffusion, but the effects of collisions with the cytoskeleton or interactions with large molecular complexes play a more important role in determining whether it can hop to an adjacent area of the membrane or not.

### Compartment size distributions

**Fig 4:**
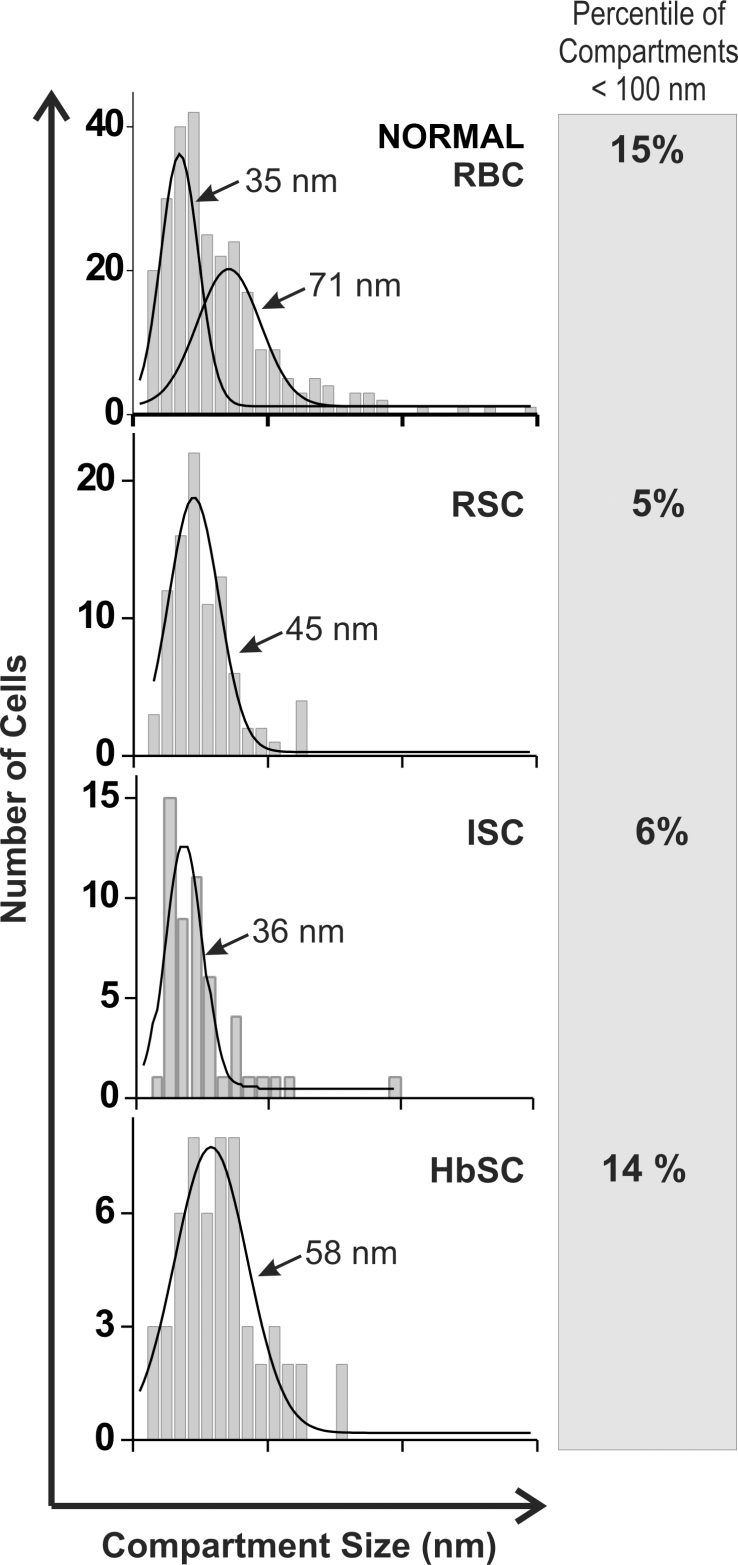
Distribution of the compartment sizes in intact healthy and sickle erythrocytes. Compartment sizes were determined by analysis of individual trajectories of labeled band 3 molecules in intact unfixed normal cells, reversibly sickled cells (RSC), irreversibly sickled cells (ISC), and HbSC cells.

The analysis of compartment size data are shown in Fig 4. A bimodal fit yielded mean compartment size values for normal RBCs of 35 and 71 nm. In normal cells, roughly half of the population diffused within an apparent compartment of 35 nm diameter, while the other half diffused within a compartment size of ~70 nm. These values are in good agreement with the estimates for thermal fluctuation of the spectrin network and the associated distances between pinning points of the cytoskeleton. The data on RSC and ISC cells yielded a single compartment size of 35–45 nm, whereas diffusion of band 3 on HbSC erythrocytes was characterized by a compartment size of 58 nm. Moreover, the percentage of compartments of sizes >100 nm decreased from normal (15%), to HbSC (14%), to ISC (6%) to RSC (5%). Based on these data, it would appear that the anion transporter experiences significantly higher barriers while diffusing in membranes of cells containing the sickle hemoglobins than in membranes from normal erythrocytes.

## Discussion

Using intact sickle erythrocytes, we have calculated the short term and long term diffusion coefficients and the spectrin compartment sizes for those band 3 molecules displaying anomalous diffusion on the surfaces of these pathologic cells. Single particle tracking of band 3 in both normal and sickle cells has revealed a bimodal distribution of D_μ_ values and a distinctive difference in the relative abundance of slow and fast populations. Both slow and fast D_μ_ values of ISC and RSC were much slower (~10x) than normal human RBCs. The loss of the band 3 population displaying rapid diffusion (~10^−10^ cm^2^/s) seen in cells with mutated hemoglobins would imply that the mobility of band 3 is hindered in these cells. In fact, ISC mobility was two times slower than RSC mobility in terms of both microscopic and macroscopic diffusion. In contrast to the HbSS cells, the short term mobility of band 3 in HbSC cells was between the slow and fast diffusing populations found in the RSC and ISC. Band 3 mobility was higher in HbSC compared to HbSS cells, perhaps relating to the fact that HbSC is a less severe hemoglobinopathy than HbSS. Moreover, the slower D_μ_ values of band 3 in sickle cells (both RSC and ISC) and HbSC cells are consistent with the fact that band 3 forms aggregates called Heinz bodies [4] in these cell types. Our data lead us to conclude that the lateral diffusion of band 3 increases in the following order: ISC< RSC< HbSC< normal cells, suggesting that the mobility of band 3 clusters roughly correlates with disease severity in erythrocytes expressing sickle hemoglobin. Whether this correlation can be eventually exploited to obtain a more molecular understanding of the disease symptoms of SCD will have to await further scrutiny.

The compartment size data would seem to suggest that the cytoskeletal network in sickle RBCs is somehow remodeled to eliminate the larger compartment size. Thus, the confined spaces in which band 3 was free to diffuse were measured at 71 and 35 nm in normal cells, but only 36 nm, 45 nm and 58 nm in ISCs, RSCs, and HbSC cells, respectively. Moreover, there were fewer very large (> 100 nm) compartments measured in sickle than normal cells. While a contraction of the spectrin network may appear to constitute the preferred interpretation of these data, we suggest that the binding and clustering of band 3 by hemichromes provides a more logical explanation of the observations. Thus, clustering of band 3 into aggregates will increase its effective size and mass, and thereby decrease its probability of hopping from one compartment to the next; i.e. thereby decreasing its D_M_ and apparent compartment size [15]. The fact that only normal cells experience a larger compartment (71 nm) of roughly twice the expected size of a spectrin corral (40 nm; [16]) suggests that only unclustered band 3 can hop a spectrin fence to move into an adjacent spectrin-enclosed corral.

In summary, our data show that the mobility of all populations of band 3 is restricted in sickle cells. The fact that band 3 on reversibly sickled cells is more mobile than on irreversibly sickled cells is consistent with previously published observations that ISC are more rigid and dense than RSC [17, 18] and that an increasingly immobile fraction of band 3 emerges with increasing sickle cell density [9]. We propose that a substantial fraction of the increased immobilization of band 3 may be related to hemichrome-induced aggregation of band 3, which can lead to either i) “pitting” of the aggregates from the membrane during transit of the RBC through the spleen, or ii) antibody- and complement-mediated recognition and removal of the pathologic erythrocyte from circulation, leading to the shortened lifespan of sickle red cells [4–6, 9, 19–22].

## Supporting Information

S1 Fig Distribution of individual diffusion coefficients of reversibly sickled cells (RSC). (TIF)

S2 Fig Distribution of individual diffusion coefficients of irreversibly sickled cells (ISC). (TIF)
